# Auxetic Structures for Tissue Engineering Scaffolds and Biomedical Devices

**DOI:** 10.3390/ma14226821

**Published:** 2021-11-12

**Authors:** Yujin Kim, Kuk Hui Son, Jin Woo Lee

**Affiliations:** 1 Department of Plastic and Reconstructive Surgery, Gil Medical Center, College of Medicine, Gachon University, 21, Namdong-daero 774 Beon-gil, Incheon 21565, Korea; pseugene@gilhospital.com; 2 Department of Thoracic and Cardiovascular Surgery, Gil Medical Center, College of Medicine, Gachon University, 21, Namdong-daero 774 Beon-gil, Incheon 21565, Korea; dr632@gilhospital.com; 3 Department of Molecular Medicine, College of Medicine, Gachon University, 155 Gaetbeol-ro, Incheon 21999, Korea

**Keywords:** auxetic, scaffold, tissue engineering, biomedical, device

## Abstract

An auxetic structure utilizing a negative Poisson’s ratio, which can expand transversally when axially expanded under tensional force, has not yet been studied in the tissue engineering and biomedical area. However, the recent advent of new technologies, such as additive manufacturing or 3D printing, has showed prospective results aimed at producing three-dimensional structures. Auxetic structures are fabricated by additive manufacturing, soft lithography, machining technology, compressed foaming, and textile fabrication using various biomaterials, including poly(ethylene glycol diacrylate), polyurethane, poly(lactic-glycolic acid), chitosan, hydroxyapatite, and using a hard material such as a silicon wafer. After fabricating the scaffold with an auxetic effect, researchers have cultured fibroblasts, osteoblasts, chondrocytes, myoblasts, and various stem cells, including mesenchymal stem cells, bone marrow stem cells, and embryonic stem cells. Additionally, they have shown new possibilities as scaffolds through tissue engineering by cell proliferation, migration, alignment, differentiation, and target tissue regeneration. In addition, auxetic structures and their unique deformation characteristics have been explored in several biomedical devices, including implants, stents, and surgical screws. Although still in the early stages, the auxetic structure, which can create mechanical properties tailored to natural tissue by changing the internal architecture of the structure, is expected to show an improved tissue reconstruction ability. In addition, continuous research at the cellular level using the auxetic micro and nano-environment could provide a breakthrough for tissue reconstruction.

## 1. Introduction

The human lifespan is increasing due to the development of medicine, improved nutrition, and an enhanced living environment; in most developed countries, the elderly population is also increasing in size. However, because the lifespan of our organs is limited, the demand for transplantation for dysfunction or damaged organs continues to increase. The number of organ donations, however, is insufficient when compared to the number of people waiting for organ transplants, causing social problems. To solve this problem, various biomedical technologies have been studied, and tissue engineering technology, which is a concept of transplanting externally produced tissues and organs to patients, is also drawing attention.

Tissue engineering aims to replace impaired tissues with new tissues or regenerated organs by combining life science and engineering [1]. Currently, tissue engineering has been used to reconstruct various biological tissues, from simple tissues, such as skin, bone, and cartilage, to complex organs such as the bladder and trachea. Three factors are essential for tissue regeneration, cells, biomolecules, and scaffolds. Among them, the scaffold plays a main role at tissue engineering by providing the structural shield for the cell survival and three-dimensional (3D) space for tissue growth [2].

Until now, various traditional scaffold fabrication techniques, such as particulate leaching, gas foaming, freeze-drying, phase separation/inversion, and fiber bonding, have been developed. However, because these methods had difficultly controlling internal architectures of the scaffold, including porosity, pore shape, and pore interconnectivity, they did not supply an optimized 3D micro-environment for cell growth and tissue generation. However, additive manufacturing, also known as 3D printing, could fabricate complex 3D structures by the deposition of materials, layer upon layer, it has recently attracted attention in the scaffold fabrication for tissue regeneration [3,4,5].

Various materials, including natural and synthetic polymers, ceramics, and metals are currently used for the production of scaffolds, but it is difficult to prepare materials with properties suitable for transplanted tissues and organs. Since the tissues that make up our body are organically combined with cells and the extracellular matrix (ECM), physical properties are inevitably different when considering artificial tissues created outside versus actual tissues inside the body. As one of the solutions to this problem, the development of a scaffold that mimics the structural characteristics of the body and can modulate mechanical characteristics, has been studied. Among them, auxetic scaffolds, which have a negative Poisson’s ratio (NPR) that can expand transversally when axially strained under tensional force, have been focused on [6,7,8,9,10,11]. In particular, many parts of the body, such as muscles, ligament tissues, vascular tissues, skin tissues, and bone tissues, are composed of negative Poisson’s ratio (auxetic) tissues, so auxetic structures have been used for tissue engineering research to replace these tissues [12,13]. In addition, auxetic structures have been used for bio-prostheses, stents, hip stems, and surgical screws because of their optimized compressive strength, shear stiffness, and elastic modulus [14,15,16,17].

In this review, we discuss the definition of the auxetic effect, the design of the structure, and the production of actual two-dimensional (2D) structures and 3D structures using various fabrication techniques, and have provided examples of tissue engineering scaffolds and biomedical devices.

## 2. Definition, Design, and Fabrication of Auxetic Structures

### 2.1. Definition of Auxetic Property

**Figure 1:**
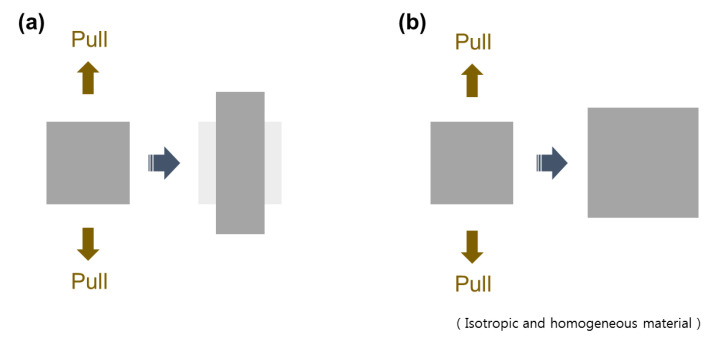
Behaviors of (**a**) conventional material and (**b**) auxetic (negative Poisson’s ratio) material.

Poisson’s ratio is the ratio of transverse expanded (or contracted) strain to longitudinal contracted (or expanded) strain in the direction of the tension (or compression) [18] (Figure 1).

Poisson’s ratio is defined as:

(1) $$\nu_{\mathrm{xy}}=-\frac{\varepsilon_{y}}{\varepsilon_{x}}$$

where ε_y_ is the transverse strain generated in response to the axial strain ε_x_ [19]. To ensure that normal materials possess a positive ratio (PPR), the coefficient (ν) was endowed to a negative sign. Generally, when a material is expanded in one direction, it contracts in the other direction perpendicular to that direction. When compressed, it expands in the other direction. However, Equation (1) was applied in a homogeneous and isotropic structure, and an additional consideration was required for heterogeneous and anisotropic structures [20,21,22]. However, some structures have a negative value, a negative Poisson’s ratio NPR or auxetic properties. In the auxetic NPR structure, transverse and longitudinal expansions are simultaneously generated by the tensional force. Additionally, by the compressional force, transverse and longitudinal contractions are simultaneously generated [23]. These special characteristic show the possibility of tissue reconstruction and replacement using auxetic scaffolds and prosthesis.

### 2.2. Design of Auxetic Structures

Until now, various auxetic structures have been studied to induce the auxetic effect. The terms of ‘re-entrant’ [24], ‘chiral’ [25], and ‘rotating’ [26] have been used according to the characteristic that causes the auxetic effect.

#### 2.2.1. Re-Entrant Unit Cells

**Figure 2:**
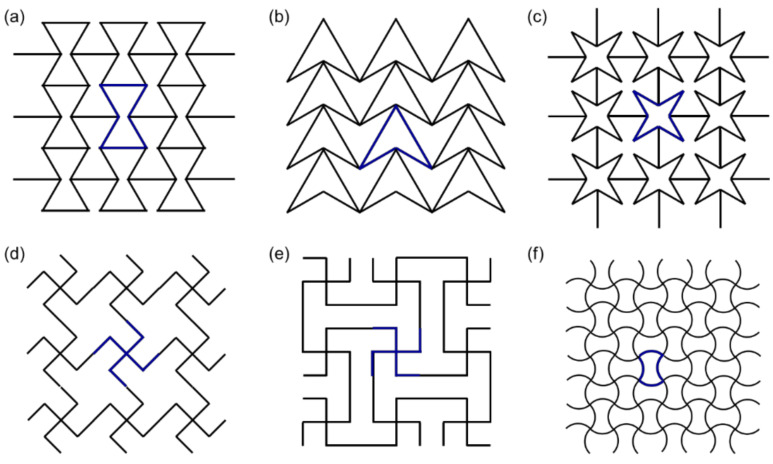
Re-entrant structures. (**a**) Re-entrant honeycomb, (**b**) double arrowhead, (**c**) star honeycomb, (**d**) structurally hexagonal re-entrant honeycomb, (**e**) lozenge grids, (**f**) sinusoidal ligaments [9].

At first, Gibson et al. [24] suggested a 2D re-entrant honeycomb structure with auxetic effects. A deformation of diagonal lines from the honeycomb created 2D re-entrant hexagons. Theoretically, by the horizontal directional load, when the diagonal ribs were stretched to the horizontal direction, they moved units along the vertical direction; thereby, they generated an auxetic effect (Figure 2a). However, actually, re-entrant honeycomb structures predominantly deform by the flexure of diagonal ribs. 

Auxetic effects are also shown in other re-entrant structures. Figure 2b,c shows that auxetic behaviors are generated by the movements of the arrowheads in the double-arrowhead structure and the rib flexure and hinging at the star honeycomb [27,28,29]. Two re-entrant structures were created by hinging the side lines of the lozenge, and square grids (Figure 2d,e). The highlighted patterns in bold are the unit cells [30,31]. The auxetic effects were generated by the expansion and rotation of the lines composed of unit cells. Another auxetic structure is a quilted structure with sinusoidal ligaments (Figure 2f) [32]. Its auxetic effect is generated by the stretching of the sinusoidal curves. In addition, the sinusoidal ligaments can be changed in response to hinged linear ligaments.

#### 2.2.2. Chiral Unit Cells

**Figure 3:**
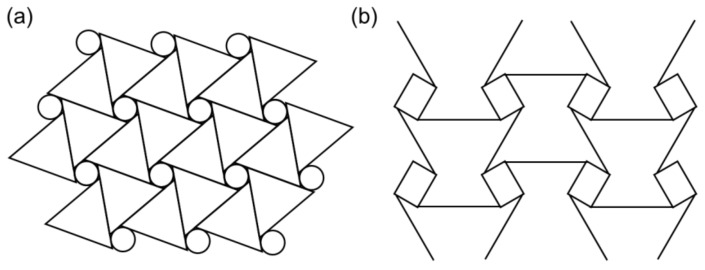
Chiral structures. (**a**) chiral structure with same units, (**b**) chiral structure with symmetrical units [9].

Chiral structures are another type of structure developed from auxetic honeycombs. As shown in Figure 3a, basic chiral units were first composed of connected straight ribs to the central nodes; these may be circles or other closed looped geometry. Then, entirely, the chiral structure was formed by the connection of all chiral units. The auxetic effects were obtained by wrapping or unwrapping the ribs by the node rotation according to an applied load. Based on this structure, Grima et al. [33] developed a structure referred to as ‘meta-chiral’ (Figure 3b). In addition, Fu et al. introduced a 3D chiral honeycomb structure by assembling chiral 2D honeycombs with four ligaments [34].

#### 2.2.3. Rotating Unit Cells 

**Figure 4:**
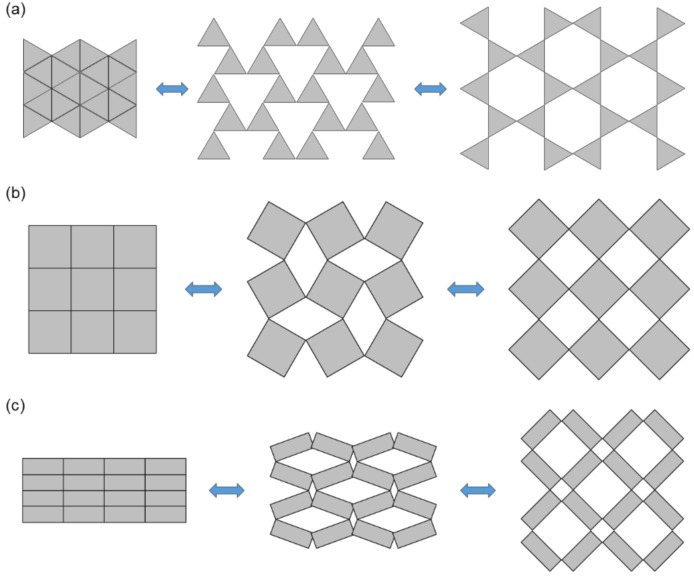
Rotating unit structures. (**a**) Triangle unit cells, (**b**) square unit cells, (**c**) rectangle unit cells [26,35,36,37]. (A figure was reproduced with permission from John Wiley and Sons [35]).

Rotating unit cell structures were designed to generate an auxetic effect in nano-structure-networked polymers and foams by the connection of triangles, rectangles, a squares that can be hinged at selected vertices (Figure 4) [26,35,36,37]. The auxetic effects were generated by the rotation of the triangles, squares, and rectangles in response to the loading. Recently, Gatt et al. [38] proposed 2D and 3D simulation models of auxetic structures based on hierarchical multilevel rotating squares.

### 2.3. Fabrication of Auxetic Structures

#### 2.3.1. Fabrication Using Additive Manufacturing

Additive manufacturing, which is also known as 3D printing or rapid prototyping, is the technology to fabricate precise 3D objects by the layer by layer deposition of materials. As its name implies, additive manufacturing accumulates a material layer to create the 3D structure. It can use digital data created by computer-aided design (CAD) software or 3D scanners. Dissimilar to traditional scaffold fabrication techniques such as particulate leaching, gas foaming, freeze-drying, and phase separation/inversion, since additive manufacturing can freely tune an internal architecture and external structure, it is expected that the production of auxetic structures for tissue engineering and medical devices could become more active. Until now, auxetic structures using additive manufacturing were utilized as scaffolds with nano and micro-environments for the cells.

Fozdar et al. [39] fabricated a three-dimensional structure using an auxetic unit cell pattern and a PEGDA (Poly(ethylene glycol) diacrylate) biocompatible polymer. They used stereolithography using digital mirror micro-devices (DMD), an additive manufacturing technology, and showed that the fabricated scaffold possessed a negative Poisson’s ratio in the micro-meter scale by the connection of unit cells. In addition, they showed hybrid patterns that properly combined positive and negative Poisson’s ratio patterns, and they produced a zero Poisson’s ratio structure through the same technology and designed unit cells [40,41]. Zhang et al. created a net-shaped soft auxetic construct with several micrometer auxetic units using a femtosecond laser and a PEGDA biocompatible polymer [42].

Easey et al. [43] introduced dome-shaped structures with auxetic behavior. They designed and fabricated 3D structures consisting of various unit cells using additive manufacturing and polylactic acid (PLA) biopolymer. The deformation of each dome structure by a compression was compared using a digital image correlation system with finite element modeling. Experimental and numerical simulations showed a close correlation and each dome composed of various unit cells presented unique indentation behavior. Wu et al. fabricated an NPR stent using fused deposition modeling (FDM) and a PLA biodegradable polymer [44]. They investigated the change in radial compressive forces by the varying thickness of the wall, diameter of the stent, and geometric parameters of NPR unit cells of the arrowhead shape. In addition, the shape memory effect of the stents was found under conditions of temperature excitation.

Ebrahimi et al. [45] suggested an anti-chiral structure with auxetic effects. When the z-directional load is applied, the circular nodes rotated and the node connected lines are bent; then, the unit cells showed an out-of-plane deformation. The Poisson’s ratios from analytical, finite element, and experimental results were similar, and their values were −1.5 < υ_xz_ < 1.

**Figure 5:**
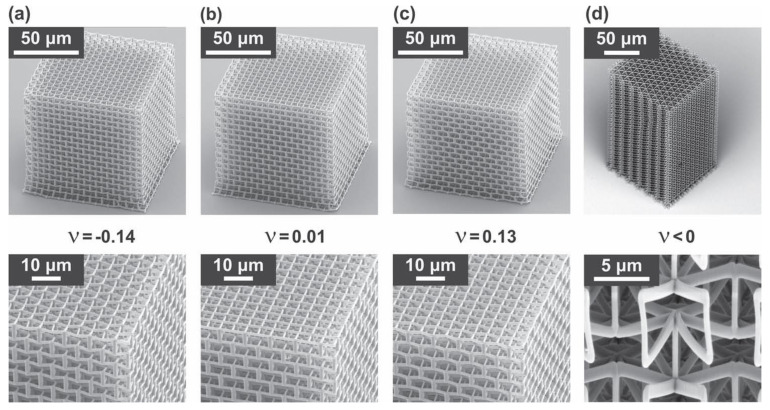
Fabrication results of the 3D meta-materials with auxetic unit cells. (**a**–**c**): 3D auxetic structure with a four-fold axis, (**d**): 3D auxetic structure with a six-fold axis. (A figure was reproduced with permission from John Wiley and Sons [46]).

Bückmann et al. [46] fabricated and characterized 3D meta-materials with submicron lines that could tailor Poisson’s ratios (Figure 5). A high resolution of their structure was realized by dip-in 3D direct laser writing lithography. Their overall size and unit size were 100 × 100 × 100 μm^3^, 10 × 10 × 10 μm^3^, and 1 × 1 × 1 μm^3^, respectively. Hengsbach et al. [47] also fabricated 3D geometry with auxetic properties by a direct laser writing process utilized on two-photon absorption with ultra-short laser pulses. Their overall size, unit size, and truss thickness were 20 × 20 × 20 µm^3^, 3 × 3 × 3 µm^3^, and 1 × 1 × 1 µm^3^, respectively. 

Yuan et al. [48] fabricated 3D auxetic structures using a selective laser sintering process and thermoplastic polyurethane (TPU) polymer. Their laser-sintered crystal structures showed a high recovery and endurance from repeating compressions. Additionally, their structure kept the auxetic property over a wide range deformations. However, their unit size was approximately 1 × 1 × 1 cm^3^, which is too large to be used in biological applications.

Although it is a two-dimensional structure, a study was also conducted to increase the scale to produce the same effect as a three-dimensional block. Remennikov et al. [49], fabricated and evaluated the performance of large-scale (approximately 190 (length) × 50 (width) × 75 (height) mm^3^) auxetic 2D blocks using nylon materials and a commercial 3D printer (Ultimaker 3 Extended, Ultimaker, Utrecht, the Netherlands). They evaluated the mechanical properties of both the experimental deformation and numerical model deformation. Park et al. [50] fabricated bio-inspired active skins for surface morphing using thermoplastic polyurethane and a similar 3D printer (Ultimaker 3, Ultimaker, Utrecht, The Netherlands).

Meanwhile, Attar et al. [51] produced an auxetic polymer graft using stereolithography-based additive manufacturing technology and the replica technique. A positive mold was fabricated by additive manufacturing, and an impression mold was produced by the replica method using alginate. Then, using an alginate-negative mold, a silicone product for a cranial prosthesis and customized skin graft was fabricated. 

#### 2.3.2. Fabrication Using Soft Lithography

Soft lithography, which is known as semiconductor fabrication technology, is the most economical and effective method to achieve an auxetic effect at a sub-micro scale. Because the lithography process uses a silicon wafer, it is difficult to create a soft structure, but it has strength for the purpose of manufacturing biosensors. In 1999, Xu et al. [52] first fabricated auxetic structures with pores of 100 µm in soft lithography, and they improved the accuracy of auxetic meta-materials. Muslija et al. [53] first fabricated an auxetic structure using a deep reactive ion-etching (DRIE) technology. Their DRIE, a soft lithography technology, showed the potential for both the precision and mass production of auxetic materials at the nanoscale. Lantada et al. [54] also fabricated a 2D auxetic structure by the combination of DRIE and precise mask production. Their procedure showed high precision, reaching nano-size, a high aspect ratio, and the possibility of promoting mass production. They manufactured cantilever structures using a re-entrant honeycomb and chiral auxetic units. Finally, they successfully cultured human mesenchymal stem cells on an auxetic scaffold to investigate cell-scaffold interactions at the sub-cellular level.

#### 2.3.3. Fabrication Using Machining Technology

Repeated geometries of rotating unit cells were cut to a 2D flat sheet to generate an auxetic effect. Therefore, auxetic structures were also produced by cutting the appropriate positions of two-dimensional structures using machining cutting technology. Bhullar et al. [55] fabricated auxetic poly (ε-caprolactone) (PCL) nanofiber membranes using electrospinning and a femtosecond laser cut technique. Their membranes had a ten times higher elasticity than a conventional PCL nanofiber membrane, and their mechanical strength could be tuned. In addition, it was also used for the purpose of stents by wrapping a 2D auxetic sheet and implementing a 3D tubular structure.

#### 2.3.4. Fabrication Using Compressed Foam

An auxetic foam was created by reorganizing the internal cellular microstructure by performing an annealing process in a compressed state [6,56]. However, because the annealing does not provide precise control over cellular microstructures, including foams, it is not easy to control a value of Poisson’s ratio. Lakes et al. first fabricated auxetic polyurethane (PU) foams using a re-entrant cell structure and temperature control with a Poisson’s ratio of −0.7 [6]. Smith et al. fabricated a missing rib-based auxetic foam using polyester polyurethane. Their final foam showed a Poisson’s ratio of −0.60 by the heating and cooling process of an initial compressed foam [30]. 

#### 2.3.5. Fabrication Using Textile Technology

Textile structures are frequently used as scaffolds or medical devices in a variety of tissue engineering applications [57,58,59]. The reason is that the textile structure created by biomaterials provides different mechanical properties from a conventional biomaterial scaffold [60]. In addition, knitted textiles are superior to woven and non-woven fabric structures, because they provide many surfaces on which cells can attach and migrate, in addition to the higher stretch obtained from interlooped yarns [60]. Various biodegradable polymers, including polycaprolactone, polylactic acid, polyglycolic acid, polyhydroxyalkanoate, and polyurethane, are spun to yarns; then, the yarns are textured. Wang et al. compared mechanical properties of auxetic warp-knitted fabrics to conventional fabrics and confirmed the better formability of auxetic fabric [61]. Ge et al. [62] theoretically analyzed a deformation characteristics of a 3D auxetic structure. Ahmed et al. [63] fabricated eight different 3D woven auxetic structures on conventional weaving machines with high-performance yarns. NPR values of the 3D woven structures ranged from −0.9 to −2.98.

## 3. Auxetic Structures as a Tissue Engineering Scaffold

### 3.1. Additive Manufacturing-Based Auxetic Scaffold

Various studies have progressed to apply auxetic structures at tissue reconstruction by utilizing additive manufacturing, which can economically produce relatively complex structures (Table 1). Soman et al. [40,41] fabricated structures combined with negative and positive Poisson’s ratio unit cells, and they fabricated zero Poisson’s ratio structures using DMD-based stereolithography and PEGDA biocompatible polymers. They cultured human bone marrow stem cells (hMSCs) to confirm a feasibility of their structures as biological scaffolds. The scaffolds did not show cytotoxicity for a culturing time of 1 week. When they compared the culture performance of negative and positive Poisson’s ratio areas, hMSCs grew mostly along the scaffold lines in the positive Poisson’s ratio area and hMSCs grew at both pores and lines of the scaffold in the positive Poisson’s ratio area [40]. 

**Figure 6:**
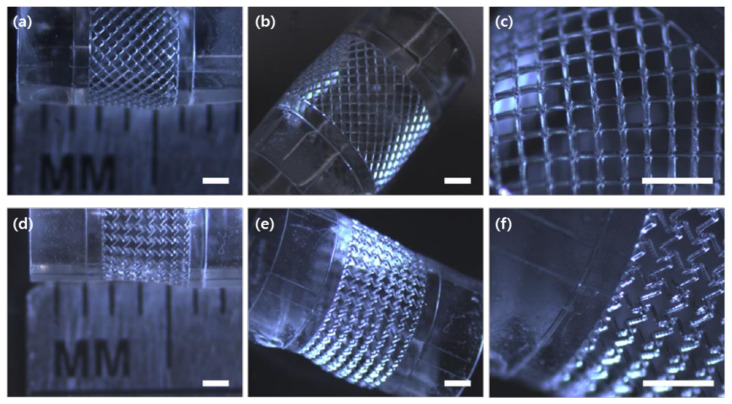
Fabrication results of tubular structures with unit cells. (**a**–**c**) Non-auxetic structure with intact ribs. (**d**–**f**) Auextic (NPR) structure with cut-missing ribs (scale: 1 mm) [65].

Warner et al. developed a multi-layered mesh scaffold with regional auxetic properties using dynamic optical projection stereolithography [64]. They had a plan to use these scaffolds for a tendon-to-muscle tissue transition. Lee et al. [65] developed a tubular scaffold with auxetic behavior using dynamic optical projection stereolithography and a sheet rolling process (Figure 6). After the rolling process, by an expansion test, the tubular scaffold exhibited a negative Poisson’s ratio. They used the biocompatible polymer PEGDA and confirmed the non-toxicity of the structure by a mesenchymal stem cell culture. Ahn et al. [12] suggested a PCL-based tubular vascular scaffold with auxetic behavior using FDM and an electrospinning process. Endothelial cells (ECs) were cultured on the inner layer composed of nanosized electrospun fibers and vascular smooth muscle cells (VSMCs) were cultivated on the outer layer with microsized electrospun fibers. The middle layer with an auxetic effect was fabricated by a 3D printer. Their auxetic scaffold exhibited a higher degree of compliance than the non-auxetic scaffold. Moreover, they proposed an optimized co-culture condition of VSMCs and ECs to inhibit a phenotype change of VSMCs.

Jin et al. [66] fabricated a 3D printing scaffold with multi-scale patterns using melt electro-writing. Patterns with thick fibers with an auxetic shape were utilized to modulate a Poisson’s ratio value and thin electrospun fibers were utilized to pack the unit cell of auxetic structures. Although they were able to only create 2D patterns, their scaffolds improved cell adhesion and growth.

Stereolithography using multiple photons was also used to create a small unit cell of several micrometers. Zhang et al. [42] used a femtosecond laser lithography and PEGDA biocompatible polymer to create a net-shaped auxetic construct and observed the behavior of cells in the structure. The unit cells of the construct were small enough to grab each cell, but it was confirmed that the cells attached to the construct showed an unusual cell division. However, there has been no in-depth discussion as to whether the control of cell migration is the Poisson’s ratio effect or the adhesion effect by the cell–structure interaction. Flamourakis et al. [67] fabricated an auxetic scaffold with re-entrant unit cells using the SZ2080 photopolymer and multi-photon lithography. They realized the real 3D architecture by accumulating unit cells of 8.6 μm; when mouse fibroblasts were seeded in the scaffold, they penetrated and proliferated in the scaffold.

Bückmann et al. [46] fabricated truly 3D meta-materials with a micron-sized re-entrant geometry by a direct laser writing optical lithography. Additionally, they demonstrated a possibility for biological cell culture studies. To exemplify these possibilities, they fabricated various Poisson’s ratio structures ranging from zero to a negative Poisson’s ratio.

The auxetic structure has also been used to develop graft materials for organ reconstruction. Ahn et al. [68] fabricated a chiral auxetic-patterned flexible 3D scaffold for the purpose of a trachea reconstruction. Thermoplastic polyurethane was used as a scaffold material to enhance the elongation ratio and rotation angle. They combined electrospun mats with a 3D-printed auxetic pattern to compensate for the poor cell-adhesive ability of polyurethane. Yu et al. [69] proposed a biomimetic trachea graft using 3D printing. A movement angle of their graft was superior to that of the native trachea. They utilized a wave pattern at the trachea scaffold to respond to human neck movements. In addition, enhancing an adhesion of bioprinted hydrogels, PCL electrospun nanofibers were assembled with a 3D-printed wave pattern. Their scaffold showed a rotational angle of 254° at the maximum (three times of a human). 

### 3.2. Machining-Based Auxetic Scaffold

**Figure 7:**
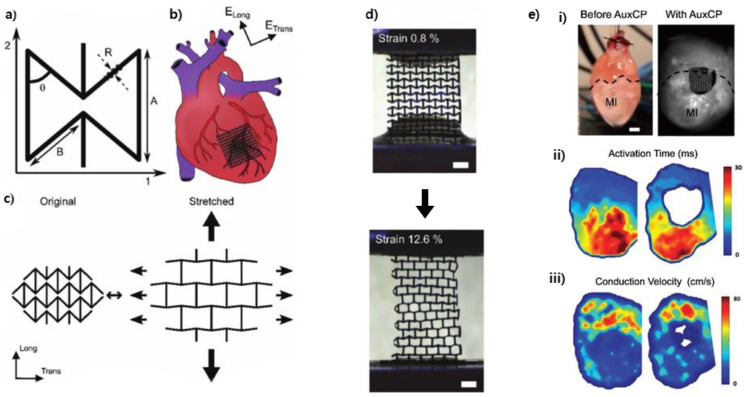
Auxetic cardiac patch for the myocardial infarction treatment. (**a**) Dimension of the re-entrant unit cell. (**b**) Schematic of the treatment using the auxetic cardiac patch (AuxCP). (**c**) Auxetic behavior by the re-entrant unit cells. (**d**) Shape change of the AuxCPs from 0.8% strain to 12.6% strain (scale bar: 1mm), (**e**) (**i**) AuxCP-treated heart (scale: 2 mm), (**ii**) Activation time maps before and after AuxCP (two weeks after treatment), (**iii**) conduction velocity maps before and after AuxCP (two weeks after treatment) [13].

Although the fabrication of complex 3D auxetic structures is challenging, a conventional machining technology can fabricate a 2D auxetic structure easily. To cure the myocardial infarction (MI), Kapnisi et al. introduced an auxetic cardiac patch using a microablation based on an excimer laser (Figure 7) [13]. They chose re-entrant unit cells as a pattern and used a chitosan–polyaniline composite as a patch material. In the in vitro experiment, the patch was cytocompatible with murine neonatal cardiomyocytes. In the in vivo experiment, their scaffold did not hinder the cardiac function and there was no fibrotic tissue generated on the patched heart two weeks after a transplantation.

### 3.3. Auxetic Foam Scaffold

An auxetic foam is created by reorganizing the internal cellular microstructure by performing an annealing process in a compressed state. Although it is difficult to freely control the internal pores, it is also applied as a tissue-engineered scaffold because it has the advantage of making bulk structures economically. Park et al. [1] fabricated a polyurethane (PU) auxetic scaffold and observed the proliferation of chondrocytes to regenerate cartilage. The Poisson’s ratio of the fabricated foam was approximately −0.4 ± 0.12. After a short-term culture (3 days), a cell proliferation on the auxetic scaffolds was 1.3 times higher than non-auxetic scaffolds. Additionally, the secreted collagen on auxetic scaffolds was 1.5 times higher than the control group. Choi et al. [70] prepared composite scaffolds of hydroxyapatite-poly (lactide-co-glycolide) (HA-PLGA: inorganic–organic composite). To induce auxetic properties, they compressed the structure with heat. Their HA-PLGA scaffold showed an enhanced osteoblast-like cell line (MG-63) proliferation than PLGA-only scaffold. Moreover, their dynamic compressive stimulation showed an improved MG-63 proliferation. They fabricated an auxetic PLGA scaffold using the same method [71]. At a static mechanical stimulation, their negative Poisson’s ratio scaffolds showed an initial improvement at cell proliferation, but after a culture time of 5 days, there was no significant difference. Their result was consistent with the osteoblast culture result of Kaspar et al. [72].

To estimate vascular differentiation from stem cells, Song et al. utilized polyurethane auxetic foams [73]. In the in vitro study, alkaline phosphatase and stem cell-like markers were lowly expressed at the auxetic scaffolds. However, vascular markers such as VE-cadherin and CD31 were more highly expressed than in the non-auxetic scaffold. Moreover, little effects were observed on the expression of the cardiac marker α-actinin. This result demonstrated the possibility of auxetic scaffolds as a vascular differentiation platform. 

Yan et al. fabricated polyurethane auxetic scaffolds with a Poisson’s ratio of −0.45 and an elastic modulus of 44 kPa to investigate the neural differentiation of stem cells [74]. In the experiment using mouse embryonic stem cells, auxetic scaffolds showed a higher cell proliferation and metabolic activity than the non-auxetic scaffold. Moreover, auxetic scaffolds induced a higher expression of β-tubulin III, a mature neuron marker, than the regular non-auxetic scaffold. This result showed that the tuning of the Poisson’s ratio of the scaffold could decide the stem cell’s fate.

### 3.4. Auxetic Textile Scaffold

When conventional textiles were shrunk in the longitudinal direction, they are expanded in the lateral direction, namely, they had a positive Poisson’s ratio. However, by realizing auxetic properties, the textile could possess enhanced properties such as a high energy absorbance and shear modulus [62]. Until now, by the combination of textile techniques, including yarns, fibers, woven, and knitted fabrics and geometries, unit cells, and various textile structures were studied [57,58,59]. Because of the variability of the knitted structure, knitting has been achieved using a superior fabrication method for NPR textiles [62]. Deshpande et al. [75] fabricated resorbable weft-knitted auxetic scaffolds with a high porosity (up to 90%) using PCL yarns. The ultimate strength of the auxetic textile scaffolds was higher than that of the native skeletal muscle tissue. The scaffold showed a good cell attachment, proliferation, and metabolic activity for 7 days.

### 3.5. Auxetic Biomedical Devices

Unique mechanical properties of auxetic structures have been of interest in biomedical applications, and several biomedical devices have been studied. Li et al. [76] created a multi-functional structure that combined the properties of both NPR structures and manufactured a shape memory alloy in one component using a selective laser melting technique. Their structure was designed and fabricated by re-entrant unit cells, and their results showed the potential for the use of these structures as bone implants. Burriesci et al. [77] registered a patent about the auxetic annuloplasty prosthesis. Because the auxetic behavior stabilized the annulus under deformation, the auxetic prosthesis would be beneficial. An auxetic prosthesis also has an advantage owing to its flexibility and fitness by the virtue of its physiological shape [78]. Martz et al. [79] proposed an artificial intervertebral disc with an isotropic negative Poisson’s ratio. As a disc material, they selected a high-density polyethylene. Additionally, they fabricated the auxetic disc using a drilling process. The disc with auxetic honeycomb unit cells could prevent the bulge that can place pressure on the lumbar intervertebral disc.

The optimization of mechanical properties such as the elastic modulus, stiffness, mobilization, and Poisson’s ratio is important to the development of bone replacement [80,81,82,83,84]. Therefore, researchers have been developing a surgical prosthesis having mechanical properties suitable for bone tissue by performing analyses, simulations, and experiments under various conditions [14,15,17,85,86,87]. Among them, meta-implant studies that can tune the mechanical properties using auxetic structure have been attracted. Kolken et al. introduced hybrid hip stems with NPR and PPR components [86,87]. To realize hybrid meta-materials, they utilized an additive manufacturing technique, namely, selective laser melting (SLM) and biomedical-grade titanium (Ti6Al4V-ELI). Their full-field strain measurements showed that the hybrid implants enhanced the bone–implant contact by the increase in the pressing side. Sanami et al. [14,15] explored the auxetic hip prosthesis for the purpose of an improved strain distribution. Recently, Ghavidelnia et al. [17] introduced a porous femoral hip meta-implant with a graded Poisson’s ratio distribution. Their implant generated a smoother stress–strain distribution, minimum areas of local stress, and a local strain concentration at the implant contact region.

Panico et al. [88] proposed a neck brace for orthopedic purposes using fractal auxetic cells. To fabricate the cervical collar, they utilized the additive manufacturing process and a polyurethane rubber-based material that would not release chemicals in the endocrine system. The geometry of the collar and the structural optimization were defined thanks to digital fabrication technologies. Finally, personalized cervical prostheses were obtained in consideration of a specific clinical case.

To fabricate the medical stent, a two-step fabrication process of auxetic sheet fabrication by laser machining and tubular form fabrication by rolling the auxetic were performed. Ali et al. [89] proposed an auxetic stent using polypropylene film for cancer treatment. At first, the auxetic sheet with rotating-square geometries using a laser cutting system was created. Additionally, the flat sheet was rolled and both edges of the sheet were welded together to form a stent. Bhullar et al. [90] also fabricated an auxetic stent to roll a polymeric sheet with auxetic geometry. Their auxetic geometry was prepared by micromachining electrospun PCL microfiber sheets. Their stent showed an improved deformation by the auxetic effect and a higher biocompatibility owing to the use of a biodegradable polymer.

Tan et al. [91] studied the relationship of the unit cell structure on radial compliance and longitudinal strain in cardiovascular stents. They compared eight stent structures with an auxetic unit cells using the finite element method. They confirmed the radial compliance to be effectively linked with longitudinal strain via Poisson’s ratio. In addition, a hybrid stent combined with negative and positive Poisson’s ratio unit cells had a low longitudinal strain. In addition, Gatt et al. [92] simulated rotating squares systems with an auxetic effect that formed a finite planar structure or a tubular conformation. It has been shown that a finite structure with a small number of repeat units has a lower young’s modulus than that of an infinite structure.

Auxetic unit cells were utilized for the development of novel surgical screws. Yao et al. [16] also developed auxetic bone screws. They fabricated tubular structures and screws with six different auxetic patterns using selected laser melting (SLM) 3D printing of Ti6Al4V powders. When they evaluated the fixation strength and mechanical properties about various types of auxetic screws, varying auxetic structures were found to alter the mechanical properties of the screw, especially its functional properties. 

Auxetic structures have also shown potential as skin grafts or active skin. Attar et al. [51] produced an auxetic polymer structure for skin grafting using a combination of stereolithography and the replica technique. Positive molds were prepared by stereolithography, and to fabricate silicone products, impression molds were utilized. It was confirmed that the fabricated structure was perfectly in close contact with the curved surface of the skull. Park et al. [50] proposed a bio-inspired skin graft for robotic gripping and surface morphing. The movement of their unit cells with star geometry showed the possibility of being a soft robotic gripper with a large-area and small-area gripping performance.

## 4. Prospect of Auxetic Structures

Research on auxetic structure has been conducted for a long time, focusing on research on the theory and simulation. Recently, as research on the human body has been actively conducted, it was confirmed that many parts of the body, such as muscles, ligament tissues, vascular tissues, skin tissues, and bone tissues, are composed of tissues with a negative Poisson’s ratio. Therefore, auxetic structures are being used in tissue engineering research to replace these tissues. Additionally, auxetic structures were utilized and expanded to the scope of the use at medical devices, including bio-prostheses, stents, hip stems, and surgical screws, owing to their enhanced compressive strength and shear stiffness. 

Currently, there are many limitations of biomaterials that can be used in the human body. Therefore, unless innovative and biocompatible materials are developed, mechanical properties matched with natural tissues must be found through a structural design. Under these circumstances, auxetic structures with tunable properties are considered to be a powerful tool that can transform limited biomaterials to be usable in our body. In addition, since they simply physically changed the internal structure, they had a high safety in vivo compared to chemical treatment. In particular, since additive manufacturing can freely tune the internal architecture and external structure, it is expected that the production of auxetic structures using additive manufacturing and their application in tissue engineering might become more active. Of course, additive manufacturing has the advantage of easily making structures with properties close to natural tissue because it can simulate structures using data prepared before 3D structure production.

Of course, until now, most of the tissue engineering studies using auxetic scaffolds were in vitro studies, and in vivo studies have not been actively conducted except for cardiac patches [56]. The reason seems that researchers focus on the auxetic effect itself rather than the tissue reconstruction. However, it is thought that there will be no problem in extending the current research results to in vivo studies. If related research continue, it is expected that it can be used in the human body in the near future. In addition, further investigations about the proliferation, differentiation, and migration of cells by the auxetic micro-environment at the cellular level could broaden the understanding of tissue reconstruction and provide a new biomedical devices.

## 5. Conclusions

An auxetic structure utilizing a negative Poisson’s ratio, which is a novel physical property different from the existing elastic modulus, hardness and strength, has not yet been closely studied. The recent utilization of new technologies, such as additive manufacturing, as well as theoretical studies, has established conditions that can actually produce a three-dimensional structure. In addition, several studies have shown new possibilities for tissue regeneration and cell proliferation, migration, and differentiation. 

The auxetic structure, which can create mechanical properties tailored to natural tissue by changing the internal architecture of the structure, is expected to show an improved tissue reconstruction ability. In the case of skin tissue or myocardial tissue, which are body tissues that undergo longitudinal expansion (contraction) by transverse expansion (contraction), vascular tissue that repeatedly contracts and expands in the radial direction by the heartbeat, a stent requiring a diameter expansion after an insertion, and orthopedic prosthesis, which must be tightly adhered to the surrounding tissue after transplantation, the auxetic effect is evaluated as an optimal solution to the problem. Additionally, recently, many biologists are turning their eyes to the 3D culture environment, recognizing the limitation that the conventional 2D cell culture based on the culture dish cannot simulate the actual tissue reconstruction environment. In such a situation, the 3D auxetic micro-environment that can simulate the motion and mechanical properties of our body well is expected to open new possibilities for tissue reconstruction.

## Figures and Tables

**Table 1:** Utilization Results of the Auxetic Structure as a Tissue Engineering Scaffold.

Author	Fabrication Technology	Specific Fabrication Method	Material	Cell Type	Biological Effect
Soman et al. [40] (2012)	Additive manufacturing	Dynamic optical projection stereolithography	PEGDA	HMSC (human bone marrow)	Grew on scaffold
Soman et al. [41] (2012)		Dynamic optical projection stereolithography	PEGDA	HMSC (human bone marrow)	Grew on scaffold
Zhang et al. [42] (2013)		Two-photon stereolithography	PEGDA	10T1/2 (mouse embryonic fibroblast)	Unable to divide
Warner et al. [64] (2017)		Dynamic optical projection stereolithography	Polyurethane	10T1/2 (Mouse embryonic fibroblast) C2C12 (mouse myoblast)	Grew on scaffold
Lee et al. [65] (2016)		Micro-stereolithography	PEGDA	HTMSC (human turbinate mesenchymal stromal cell)	Proliferation increased on NPR
Jin et al. [66] (2021)		Fused deposition modelling	poly(ε-caprolactone)	HUVEC (human umbilical vein endothelial cell) BMSC (bone marrow stem cell)	Adhered and grew on scaffold
Ahn et al. [68] (2019)		Fused deposition modelling + electrospinning	Thermoplastic Polyurethane	HTMSC (human turbinate mesenchymal stromal cell)	Grew on scaffold
Ahn et al. [12] (2019)	Additive manufacturing + electrospinning	Fused deposition modelling	poly(ε-caprolactone)	HUVEC (human umbilical vein endothelial cell) VSMC (vascular smooth muscle cell)	Made multi-layers
Yu et al. [69] (2021)		Fused deposition modelling + electrospinning	poly(ε-caprolactone)	-	Attached with the printed scaffold and hydrogel
Muslija et al. [53] (2021)	Soft lithography	Deep reactive ion etching	Silicon	-	-
Lantada et al. [54] (2015)		Deep reactive ion etching	Silicon	HMSC (human mesenchymal stem cell)	Interacted at a sub-cellular level
Kapnisi et al. [13] (2018)	Machining	Micro-ablation	Chitosan (polyaniline coating)	Neonatal rat ventricular myocytes and fibroblasts	Grew on scaffold (cytocompatibility)
Park et al. [1] (2013)	Foaming	Compressed foams	Polyurethane	Chondrocytes (primary from cartilage)	Proliferation increased
Choi et al. [70] (2016)		Solvent casting/salt leaching	HA/PLGA	MG-63 (human Osteoblast)	Proliferation increased
Choi et al. [71] (2016)		Solvent casting/salt leaching	PLGA	MG-63 (human Osteoblast)	Proliferation increased
Song et al. [73] (2018)		Heated foams	Polyurethane and polyester	ES-D3 (mouse embryonic stem cell) iPSK3 (human induced pluripotent stem cell)	Vascular differentiation increased
Yan et al. [74] (2017)		Compressed and heated foams	Polyurethane	ES-D3 (mouse embryonic stem cell) iPSK3 (human induced pluripotent stem cell)	Neural differentiation increased
Deshpande et al. [75] (2020)	Textile	Fabric knitting	poly(ε-caprolactone)	Human dermal fibroblasts	Cell metabolic activity increased

## Data Availability

Not applicable.
